# Validation of a prefractionation method followed by two-dimensional electrophoresis – Applied to cerebrospinal fluid proteins from frontotemporal dementia patients

**DOI:** 10.1186/1477-5956-2-7

**Published:** 2004-11-18

**Authors:** Sara Folkesson Hansson, Maja Puchades, Kaj Blennow, Magnus Sjögren, Pia Davidsson

**Affiliations:** 1 Department of Clinical Neuroscience, Unit of Experimental Neuroscience, The Sahlgrenska Academy at Göteborg University, Sahlgrenska University Hospital/Mölndal, S-431 80 Mölndal, Sweden; 2 Department of Clinical Science, AstraZeneca R&D, Södertälje, Sweden; 3 Department of Experimental Medicine/Molecular Science, AstraZeneca R&D, Mölndal, Sweden

## Abstract

**Background:**

The aim of this study was firstly, to improve and validate a cerebrospinal fluid (CSF) prefractionation method followed by two-dimensional electrophoresis (2-DE) and secondly, using this strategy to investigate differences between the CSF proteome of frontotemporal dementia (FTD) patients and controls. From each subject three ml of CSF was prefractionated using liquid phase isoelectric focusing prior to 2-DE.

**Results:**

With respect to protein recovery and purification potential, ethanol precipitation of the prefractionated CSF sample was found superior, after testing several sample preparation methods.

The reproducibility of prefractionated CSF analyzed on 2-D gels was comparable to direct 2-DE analysis of CSF. The protein spots on the prefractionated 2-D gels had an increased intensity, indicating a higher protein concentration, compared to direct 2-D gels. Prefractionated 2-DE analysis of FTD and control CSF showed that 26 protein spots were changed at least two fold. Using mass spectrometry, 13 of these protein spots were identified, including retinol-binding protein, Zn-α-2-glycoprotein, proapolipoproteinA1, β-2-microglobulin, transthyretin, albumin and alloalbumin.

**Conclusion:**

The results suggest that the prefractionated 2-DE method can be useful for enrichment of CSF proteins and may provide a new tool to investigate the pathology of neurodegenerative diseases. This study confirmed reduced levels of retinol-binding protein and revealed some new biomarker candidates for FTD.

## Background

Frontotemporal dementia (FTD) accounts for up to 20% of presenil dementia cases [1] and is, after Alzheimer's disease (AD), the second most common form of early onset dementia (at age < 65 years) [2]. The clinical picture in FTD is characterized mainly by changes in personality and social behavior, signs of disinhibition, lack of insight and changes in eating preferences [1]. Memory disturbances, which prevail in AD, may also be found in FTD but not usually to the same extent [3]. Post-mortem pathological examination reveals bilateral atrophy of the frontal and anterior temporal lobes in FTD and the ventricular system is sometimes widened frontally [4]. The histological findings provide a basis for the division of FTD into various subtypes. Neurofibrillary tangles, a prominent neuronal accumulation of hyperphosphorylated and filamentous forms of the microtubule associated protein tau, are found in FTD with Parkinsonism linked to chromosome 17, the hereditary variant of FTD, caused by mutations in the tau gene [5]. Another FTD variant, Pick's disease, is characterized by the presence of neuronal inclusion bodies called Pick bodies, containing filamentous tau and ubiquitin aggregates. The most common type of FTD is frontal lobe degeneration of non-Alzheimer type, which is clinically indistinguishable from Pick's disease, and histologically characterized by neuron loss and gliosis in the absence of distinctive histopathology, such as neurofibrillary tangles or other intracellular inclusions [4].

The diagnosis of FTD is often difficult and would be greatly enhanced by the use of disease specific neurochemical markers [6]. Several neuro-specific proteins in the cerebrospinal fluid (CSF) of FTD have been investigated [7,8] and elevation of cytoskeleton markers such as neurofilament light protein and tau have been found [7-10]. In order to expand the search for diagnostic biomarkers, which would also lead to a better understanding of the pathophysiological mechanisms of neurodegeneration, two-dimensional electrophoresis (2-DE) investigations of the CSF have previously been performed [11-15]. 2-DE can effectively separate several proteins and their isoforms simultaneously and is a useful tool for identifying quantitative and qualitative protein differences between the diseased and normal state. Previous proteomic studies by our group have shown for the first time that several proteins involved in FTD pathology are not effected in the CSF of AD patients and vise versa, thus establishing a likely difference in the pathophysiological mechanism between FTD and AD [11,12].

Some abundant proteins, for example albumin and immunoglobulins, limit the total amount of CSF proteins that can be loaded on the 2-D gel, resulting in difficulties detecting low abundant proteins of CSF. By using liquid phase isoelectric focusing (LP-IEF) as a prefractionation step prior to 2-DE we have previously shown that less abundant CSF proteins can be enriched, thus making them more easily detected and identified by mass spectrometry (MS) [16]. The advantage of this method is that a larger volume of CSF (3 ml) can be used as starting material and that proteins outside the selected pH interval of the 2-D gel can be excluded. For several years alternative prefractionation methods prior to 2-DE have been reported [17-22], each with its advantages and disadvantages.

The aim of the present study was to improve the prefractionation procedure for individual CSF samples and to determine its reproducibility. Moreover, the second aim of this study was to apply the method and further explore disease-influenced proteins in CSF from FTD patients compared to controls.

## Results

### Preparation of prefractionated CSF samples prior to 2-DE

**Figure 1:**
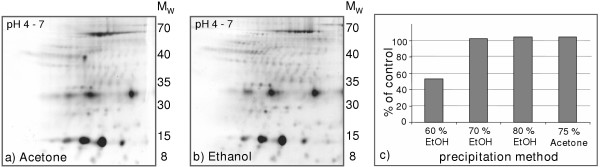
**Comparison of different protein precipitation methods**. a) 75% acetone precipitation and b) 70% ethanol precipitation of prefractionated SYPRO Ruby stained CSF proteins with pH 4.5–6.0, separated on IPG-strips, pH 4–7. The molecular weights (M_W_) are in kDa. c) The protein recovery as % of control (untreated CSF) after precipitation with indicated concentrations of ethanol and 75% acetone. The protein recovery was measured using the RC DC protein assay (Bio-Rad).

Several strategies to reduce impurities, i.e. salt and ampholytes, of the prefractionated CSF samples were performed including, trichloroacetic acid (TCA)-acetone precipitation, ethanol precipitation, chloroform/methanol/water precipitation and, micro Bio-Spin desalting column. The protein pattern on the 2-D gels and protein recovery from the different sample preparation methods were compared to that of acetone precipitation, previously used prior to prefractionated 2-DE of CSF [16]. We found that ethanol precipitation gave the best result and allowed us to reduce the focusing time from 25 000 Vh to 20 000 Vh, as recommended by the Protean cell manufacturer. Evaluation of gels with ethanol precipitated sample showed that the same protein spots were present, or even a few more, with similar intensities as in the gels with the acetone treated CSF-samples (figure 1a and 1b). Furthermore, the optimal ethanol concentration was tested showing that a concentration of at least 70% ethanol was sufficient to generate a total protein recovery, determined by the RC DC protein assay (figure 1c). An ethanol concentration of 71.25% (3/4 95% ethanol and 1/4 sample) was used in the subsequent studies.

Both TCA-acetone and chloroform/methanol/water precipitation substantially reduced the number of spots and resulted in streaky 2-D gels (data not shown). The use of biospin columns gave well-focused 2-D gels but several protein spots were reduced or lost (data not shown).

### Reproducibility of prefractionated and unfractionated CSF on 2-D gels

**Figure 2:**
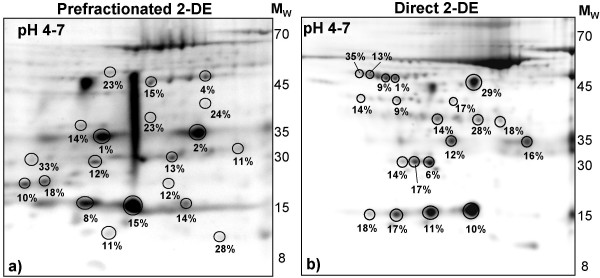
**Reproducibility study of direct and prefractionated 2-DE of CSF on SYPRO Ruby stained 2-D gels**. a) Represents a standard 2-D gel image of prefractionated (using LP-IEF) CSF fractions with pH 4.5–6.0, separated on a pH 4–7 IPG-strip. Numbers represents the CVs of encircled protein spots from four individually prefractionated, identical CSF samples separated by 2-DE. The mean CV of the 20 marked spots is 14.6% ± 7.9% (mean ± SD). b) Represents a standard 2-D gel image of directly analysed (after acetone precipitation) CSF proteins, separated on a pH 4–7 IPG-strip. Numbers represents the CVs of encircled protein spots from four replicate gels. The mean CV of the 20 marked spots is 15.4% ± 8.3% (mean ± SD). Molecular weights (M_W_) are in kDa.

Four identical CSF samples were individually prefractionated by LP-IEF. Fractions 6–9 (having a pH of 4.5–6.0) were pooled and analyzed on pH 4–7 strips in four replicates. Coefficients of variation (CVs) were calculated for the protein spots quantities, determined by the PD-Quest software, and a selection of 20 spots had CVs ranging from 1–33% (15.4% ± 8.3%, mean ± SD) (Figure 2a). The spots were selected to represent a broad range of proteins present in each replicate, i.e. proteins of different molecular weights and isoelectric points (pIs), low, medium and high abundant proteins as well as different isoforms of the same protein. The reproducibility of direct 2-DE of CSF, using pH 4–7 gels, in four replicates was also determined and the CVs of a similar selection of 20 spots ranged from 1–35% (14.6% ± 7.9%, mean ± SD) (Figure 2b).

### Comparison of direct and prefractionated 2-DE

**Figure 3:**
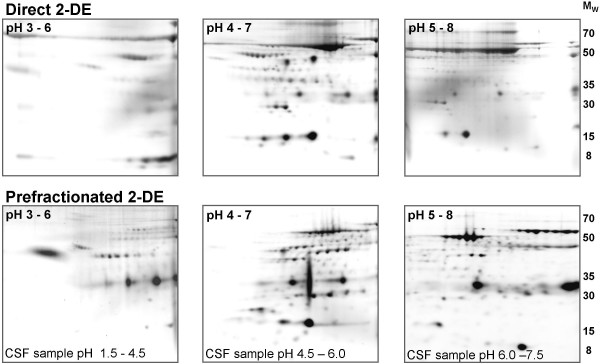
**Comparison of direct and prefractionated CSF on SYPRO Ruby stained 2-D gels**. The upper figures represents standard images of direct 2-DE, and the lower figures represents prefractionated 2-DE. The pH interval of the IPG strips is denoted in the upper left corner of the gels and the pH range of the prefractionated CSF samples at the bottom of the gel images. Molecular weights (M_W_) are in kDa.

Comparison of direct and prefractionated 2-DE showed that the prefractionated gels contained more spots with a higher protein concentration (Figure 3). The spots in the pH 3–6 and pH 5–8 gels in particular were increased in both number and density after prefractionation.

### A proteomic study comparing prefractionated CSF from FTD patients and control subjects

**Figure 4:**
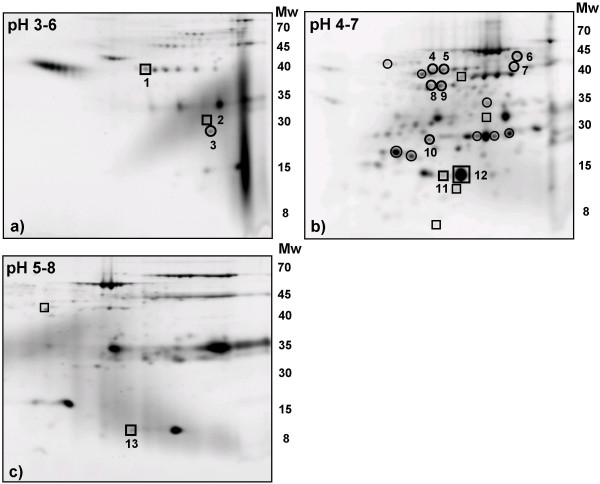
**Prefractionated CSF from FTD patients compared to non-demented controls**. Protein densities increased (squares) or decreased (circles) at least two times in prefractionated FTD CSF, analyzed using SYPRO-Ruby stained 2-DE gels. The five FTD patients were 70.6 ± 5.6 (mean ± SD) year-of-age and the five non-demented controls were 59.2 ± 11.9 (mean ± SD) year-of-age. The numbers on the 2-D gel pattern correlate each identified protein to the data given in table 2. Molecular weights (M_W_) are in kDa. a) Fraction 2–5 with pH 1.5–4.5 was analysed using a pH 3–6 linear IPG-strip. b) Fraction 6–9 with pH 4.5–6.0 was analysed using pH 4–7 linear IPG-strip. c) Fraction 10–14 with pH 6.0–7.5 was analysed using pH 5–8 linear IPG-strip in the first dimension.

The prefractionated 2-DE method was used to screen for changes in the CSF proteome of five FTD patients compared to that of five non-dementia controls in the pH intervals 3–6, 4–7, and 5–8 (Figure 4a,4b,4c).

Comparing the protein densities of the gels, 10 spots were up regulated and 16 spots down regulated, at least two fold, in FTD patients compared to non-dementia controls. Increased proteins are marked with a square and decreased with a circle (Figure 4a,4b,4c). Thirteen of the protein spots, corresponding to 7 different proteins, were identified by mass spectrometry (MS) Table 1). In the FTD group the following protein spots were increased: One isoform of Zn-α-2-glycoprotein (ZAG), proapolipoproteinA1 (ProapoA1), β-2-microglobulin (β-2-m) and two isoforms of transthyretin (TTR), while a reduction was seen in four isoforms of serum albumin, two isoforms of alloalbumin and retinol binding protein (RBP), compared to controls (Table 1).

**Table 1:** CSF proteins increased or decreased, at least two fold in FTD vs. control

***Spot no.***	***Protein identity***	***NCBI Acc. no.***	***Theor. Mw (kDa)***	***Theor.pI***	***No. peptides matched***	***Seq. cov. (%)***	***Levels in FTD vs. control***	***FTD spot norm. density (mean ± SD)***	***Control spot norm. density (mean ± SD)***
**1**	Zn-α-2-glycoprotein	141596	31.6	5.70	4	22	↑	42256 ± 7227	4189 ± 979
**2**	proapolipo- protein A1	178775	28.9	5.45	14	43	↑	11572 ± 10432	4575 ± 2309
**3**	retinol- binding protein	20141667	20.9	5.27	4	35	↓	3680 ± 1675	8131 ± 4857
**4**	serum albumin	113576	52.0	5.69	11	22	↓	1819 ± 1657	7272 ± 1812
**5**	serum albumin	113576	52.0	5.69	11	22	↓	2415 ± 964	7839 ± 1906
**6**	serum albumin	113576	66.0	5.69	12	20	↓	1003 ± 309	3098 ± 1428
**7**	serum albumin	113576	66.0	5.69	9	15	↓	1528 ± 587	4896 ± 2551
**8**	alloalbumin	178345	69.2	5.99	12	19	↓	1361 ± 530	7454 ± 1748
**9**	alloalbumin	178345	69.2	5.99	12	19	↓	2463 ± 806	8395 ± 1475
**10**	retinol-binding protein	20141667	20.9	5.27	4	35	↓	1744 ± 551	5871 ± 2040
**11**	transthyretin	339685	13.8	5.3	8	81	↑	45198 ± 26202	9149 ± 2685
**12**	transthyretin	339685	13.8	5.3	8	81	↑	303002 ± 72750	147732 ± 30928
**13**	β-2-microglobulin	4757826	12.9	5.77	5	46	↑	41938 ± 43510	12287 ± 3161

The proteins were identified by MALDI-MS. The spot numbers refer to those given in figure 4.

## Discussion

In this study we present an improved method for increased detection of CSF proteins by a combination of LP-IEF and 2-DE, followed by SYPRO Ruby protein staining and protein identification by mass spectrometry, for investigation of protein differences in CSF of FTD patients compared to controls. To our knowledge no other prefractionation method combined with 2-DE has so far been developed and evaluated for CSF proteins.

The study showed that the reproducibility of prefractionated 2-D gels could be compared to that of direct 2-D gels, indicating that the extra prefractionation step did not introduce additional variation and could be reproduced from sample to sample. The protein detection and quantitative reproducibility of Coomassie Brilliant Blue [23], silver, [24,25] and SYPRO Ruby [26,27] staining of direct 2-DE gels has previously been described. In one SYPRO Ruby study [26] the reproducibility of the quantification of 20 proteins, selected to represent well matched proteins of different molecular weight and intensity, from four replicate gels, had CVs ranging from 3 to 33%. This is in agreement with our findings using a similar selection of 20 proteins, where the prefractionated 2-DE CVs ranged from 1–33% (mean 14.5%) and the direct 2-DE ranged from 1–35% (mean 15.4%). Mainly very faint spots have CVs in the higher range (Figure 2).

In addition to the high salt concentration of CSF (> 150 mmol/L), ampholytes are also introduced into the sample in the prefractionation step, LP-IEF. We previously reported that the focusing time in the first dimension of prefractionated 2-DE had to be increased [16] probably due to insufficient "clean up" of the sample by acetone precipitation. Therefore, different "clean up" procedures were tested. Precipitation using ethanol was found to be most effective, keeping the number and intensity of the protein spots constant and allowing us to reduce the focusing time in the first dimension. We found that TCA-acetone precipitation reduced the protein content of the sample in agreement with a study of directly analyzed CSF samples [13]. In contrast to the results of Yuan et al. [13] a substantial loss of protein spots using the Bio-Spin column was found. The reason might be that proteins are retained in the spin column to a higher degree in the presence of ampholytes (Servalytes), which are small charged peptides. Ethanol precipitation of plasma samples has previously been performed[28], showing that a concentration of 66.6% ethanol was sufficient to precipitate 99% of the proteins [28]. This is in agreement with our findings, that 100% of CSF proteins are precipitated at ethanol concentrations above 70%.

When comparing direct and prefractionated 2-DE it is evident that the prefractionated gels contain more spots, with higher protein quantities. Thus, the CSF proteins are enriched in the prefractionation step, simplifying their identification by MS, as shown in our previous study [16]. CSF analysis on the pH 3–6 and pH 5–8 gels in particular is improved by the prefractionation step, probably because the amount of CSF proteins in these pH ranges, without prefractionation, is rather low.

In order to widen our search for protein differences in the CSF of FTD patients the improved prefractionated 2-DE procedure was applied to CSF from five FTD patients and five control subjects. 26 protein spots were changed at least two fold and 13 of these protein spots, representing seven different proteins, were identified as ZAG, ProapoA1, β-2-m, TTR, RBP, serum albumin and alloalbumin. Our previous direct 2-DE study [12] of the FTD proteome showed that 7 proteins were significantly altered compared to controls, including granin like neuroendocrine precursor, apolipoprotein E, pigment epithelium derived factor, RBP, haptoglobulin and albumin.

A reduced level of RBP was consistent between our two studies, and in this case RBP was found reduced in both the pH 4–7 and the pH 5–8 gels. In contrast, CSF analysis of AD showed increased levels of one isoform of RBP [11], indicating a different role of RBP in the pathology of AD and FTD. RBP is synthesized by hepatic parenchymal cells, after binding to its ligand retinol, the complex is secreted into the circulation [29], where it further complexes with the plasma protein TTR. CSF RBP concentration has been shown to correlate to those of serum [30]. Serum RBP and retinol have been found to be reduced during acute infection and the decrease is proportional to the extent of the infection [31], suggesting that reduced RBP levels may result from an inflammation in the FTD brain.

Brain TTR is exclusively produced, secreted and regulated by the choroid plexus [32-34]. TTR makes up 25% of the total CSF protein content [32] and even higher concentrations exist during prenatal and early postnatal life, indicating an importance of the protein in CNS development [35]. In this study, the levels of two isoforms of TTR were increased in the CSF from FTD patients. To our knowledge, the TTR levels of FTD CSF have not previously been studied, but in AD the CSF levels were decreased in an immunological study, not differentiating between TTR isoforms [36]. In contrast, the direct 2-DE study of the AD proteome [11] showed an increased level of TTR, but of a more acidic isoform, compared to this study. This highlights the capacity of 2-DE to quantify specific isoforms.

One isoform of β-2-m was found increased in this study. Other studies have shown that CSF β-2-m is elevated in patients with various neurological diseases including AD [11], infectious meningoencephalitis [37], neurosarcoidosis [38] and AIDS dementia complex [39]. β-2-m constitutes the non-covalently bound light chain of major histocompatibility complex class I molecule (MHCI) [40]. The MHCI complex is expressed on the surface of all nucleated cells and the association of β-2-m to the MHCI transmembranal chain is an absolute requirement for the antigenic presentation function of the complex [40]. It has been proposed that conformational changes of the MHCI complex, associated with cell injury, can be responsible for increased shedding of β-2-m from the cell membranes with consequent expansion of the circulating β-2-m pool [41].

The function of ZAG is unknown but studies have shown that it is present in several body fluids, including CSF, sweat, saliva, seminal fluid, plasma, milk, amniotic fluid and urine, suggesting a fairly widespread exocrine function of the protein [42]. In this study increased levels of ZAG were found in FTD CSF and to our knowledge, ZAG has not previously been associated with dementia.

The level of ProapoA1 was also increased in this study. Our previous study of the FTD proteome [12] did not show any increase in ProapoA1. Nevertheless, our study of the AD proteome [11] detected reduced levels of 3 isoforms of ProapoA1.

The reason that several protein changes were inconsistent between this and our previous study of the FTD proteome may be explained by the fact that a smaller sample size and a different population of FTD patients, which is a rather heterogeneous disease, were used in this study. Due to the small sample size it must also be emphasized that the protein changes found in this study are preliminary. Moreover, direct and prefractionated 2-DE are still two different proteomic approaches and a somewhat different analytical window was not unexpected. Indeed, direct and prefractionated 2-D gels show different protein patterns, for example, the apolipoprotein E and apolipoprotein J isoforms seem to be missing in the prefractionated 2-D gels, which may be explained by the fact that lipoproteins tend to adhere to plastics [43] and could be lost during LP-IEF or additional sample transfer steps in the prefractionation procedure. However, the lipoprotein, ProapoA1 could still be detected in the prefractionated 2-D gels. The proteins most likely to be favored by a prefractionation step are low abundant hydrophilic proteins, which most likely are present in CSF. Nevertheless, this investigation of the FTD proteome failed to detect any very low abundant brain specific proteins.

As shown in the present study, the levels of specific isoforms are altered and these are unlikely to be detected using methods measuring the total concentration of a protein. Therefore, determination of posttranslational modifications is of importance for understanding the neuropathology in FTD, and 2-DE is a useful method for sensitive detection of different protein isoforms.

## Conclusions

We have shown that the prefractionated 2-DE method is reproducible to the same extent as traditional 2-DE and can enrich CSF proteins in the gel. This approach may offer new perspectives on the pathology of neurodegenerative diseases. Prefractionated 2-DE analysis of FTD CSF proteins confirmed some of the proteins previously detected by direct 2-DE and revealed some new biomarker candidates. The protein changes should be further validated on a larger patient material, preferably also with complementary methods, in order to assess any of the proteins potential as biomarkers for FTD.

## Methods

### CSF samples

CSF samples were obtained from the Clinical Neurochemical Laboratory (Sahlgrenska University Hospital/ Mölndal). All CSF samples had a normal white-cell count, normal blood-brain barrier function and absence of intrathecal IgG and IgM production. The CSF samples for the proteomic study, described in table 2, were obtained from 5 FTD patients aged 70.6 ± 5.6 (mean ± SD) years and 5 non-dementia controls aged 59.2 ± 11.9 years (mean ± SD). FTD was diagnosed according to the Lund Manchester criteria [4]. The severity of dementia was evaluated using the Mini Mental State Examination (MMSE) [44]. The control group, "non-demented controls" consisted of subjects with minor psychiatric complaints or subjective memory complaints that could not be verified by clinical examination, CSF analysis or neuropsychological testing. All control individuals had MMSE scores of 29–30. Lumbar puncture was performed in the L4–L5 vertebral interspace. The first 12 mL of CSF was collected and gently mixed to avoid possible gradient effects. The CSF samples were then centrifuged at 2,000 g for 10 min to eliminate cells and other insoluble material, and stored at -80°C.

**Table 2:** CSF samples included in the prefractionated 2-DE study

***Subject***	***Age^a)^***	***Sex***	***Albumin ratio^b)^***	***Tau (ng/L)***	***Aβ (ng/L)***	***MMSE***
**FTD 1**	64	F	6.3	633	779	20
**FTD 2**	72	F	6.7	364	668	22
**FTD 3**	73	M	4.1	409	245	15
**FTD 4**	78	F	8.0	283	833	10
**FTD 5**	66	F	6.9	302	642	19
**Control 1**	63	M	6.7	368	599	29
**Control 2**	63	F	8.7	274	1070	30
**Control 3**	74	M	6.4	270	412	29
**Control 4**	54	F	5.5	210	1160	30
**Control 5**	42	M	2.8	318	1620	29

a) The age of the FTD group is 70.6 ± 5.6 years (mean ± SD) and the age of the control group is 59.2 ± 11.9 years (mean ± SD)

b) [CSF albumin(mg/L)] / [serum albumin(g/L)]

The study was approved by the Ethical Committee of Göteborg University. All participants or their relatives gave their informed consent to participation in the study, which was performed in accordance with the Declaration of Helsinki.

### Purification and precipitation methods

To find a method for effective reduction of impurities and maximal protein recovery, 300 μL of prefractionated pooled CSF samples (fraction 6–9, pH 4.5–6.0) were purified using each of the following methods:

1. *Ice cold acetone precipitation*; acetone: sample (4:1, v/v) precipitated at -20°C for 2 hours.

2. *Ice cold acetone-TCA precipitation*; 2a) acetone: TCA: sample (4:10%:1, v/w/v) at -20°C for 45 min. 2b) acetone: TCA: sample (4:20%:1, v/w/v) at -20°C for 45 min. The protein pellet was washed 2 times with acetone after centrifugation.

3. *Chloroform/methanol/water precipitation*, chloroform: methanol: sample (4:8:3, v/v/v) at room temperature for 2 hours.

4. *Ice cold ethanol precipitation*; final concentrations of 60%, 70% and 80% ethanol was added to the sample and precipitated for 2 hours at -20°C.

5. *Purification using micro Bio-Spin column *(Bio-Rad, Hercules, CA, USA) with a M_W _cut off of 6 kDa. The purification procedure w two-dimensional electrophoresis (2-DE) as performed according to the manufacturer's instructions.

After precipitation all samples were centrifuged and the protein pellet analyzed on 2-D gels, described below.

The protein recovery of acetone and ethanol treated samples was measured using the RC DC protein assay (Bio-Rad) according to the manufacturer's instructions.

### Prefractionation, sample preparation and 2-DE procedure

The CSF samples from individual patients were prefractionated using LP-IEF in the Rotofor cell (Bio-Rad). Three mL CSF sample was mixed with 9 mL millipore water, 1% ampholytes (Servalyte pH range 3–10, Serva Electrophoresis, GmbH, Germany), 20 mM dithiothreitol (DTT) and 1 × Complete antiprotease solution (Roche Diagnostics, Mannheim, Germany). The focusing was performed at 4°C and at 12 W constant power for 2.5 hours. Then the 20 Rotofor fractions were harvested and fraction 2–5 corresponding to pH 1.5–4.5, fraction 6–9 corresponding to pH 4.5–6.0 and fraction 10–14 corresponding to pH 6.0–7.5 were pooled and concentrated to 300 μL in a vacuum centrifuge prior to 2-DE.

In the FTD-study, the prefractionated pooled protein fractions were precipitated using 900 μL 95 % ice-cold ethanol (71.25% final conc. ethanol) for two hours at -20°C. The mixture was centrifuged at 10,000 × g for 10 min at 4°C. The protein pellets were air-dried and then resolved in a buffer containing 9 M urea, 35 mM tris, 42 mM DTT, 2% 3-((3-cholamidopropyl) dimethylammonio)-1-propanesulfonate (CHAPS), 0.66% sodium dodecyl sulfate (SDS), 2% IPG buffer and bromophenol blue.

The first dimension was carried out using immobilized pH gradient (IPG) strips (Bio-Rad), 7 cm, pH 3–6 for Rotofor fractions 2–5, pH 4–7 for Rotofor fractions 6–9 or pH 5–8 for Rotofor fractions 10–14. The IPG-strips were actively rehydrated in the CSF-protein sample for 12 h at 50 V followed by protein focusing for 20,000 Vh using the Protean IEF Cell (Bio-Rad). The IPG strips were placed in 5 ml equilibration solution (50 mM Tris-HCl pH 8.8, 6 M urea, 30% glycerol, 2% SDS, bromophenol blue) containing 1% DTT, and 2.5% iodoacetamide in the second equilibrium step for 2 × 15 min.

The second dimension was performed using the Nu-PAGE gel system (NOVEX, San Diego, CA, USA) with (2-(N-morpholino) ethane sulfonic acid (MES) buffer: 50 mM MES, 50 mM tris, 3.5 mM SDS, 1 mM EDTA), for 35 min at 200 V.

In the direct 2-DE procedure, 300 μL CSF proteins were precipitated using 900 μL ice-cold acetone and stored for two hours at -20°C. The mixture was then centrifuged at 10,000 × g for 10 min at 4°C. The 2-DE procedure was performed as described above for prefractionated 2-DE.

### Visualisation and evaluation

The gels were stained using SYPRO Ruby Protein Stain (Molecular-Probes, Eugene, Oregon, USA) according to the manufacturer's instructions. Image acquisition and analysis were performed on a Fluor-S MultiImager (Bio-Rad). The protein spots were detected, quantified and matched with the PD-Quest 2-D gel analysis software, v.7.0 (Bio-Rad). The gels were normalized according to the total quantity in valid spots (the raw quantity of each spot in a member gel is divided by the total quantity of all the spots in that gel that have been included in the Master gel). Protein levels increased or decreased two fold were taken into account.

### In-gel tryptic digestion and sample preparation

The protein digestion method has been previously described in detail [11]. Briefly, the gel pieces were digested with porcine trypsin (Promega Corporation, Madison, USA) and the peptides were extracted with formic acid (FA) and acetonitrile (ACN). The digested protein sample was dried under vacuum and then dissolved in 10 μL 0.2% triflouroacetic acid (TFA) (v/v). The samples were applied to the MS probe using the AnchorChip™ technology (Bruker daltonics, Bremen, Germany) as previously described [45]. Briefly α-cyano-4-hydroxy-cinnamic acid (CHCA) solution (100 g/L in 90% acetone, 0.005 % TFA) was spread out evenly on the sample plate surface creating the CHCA matrix layer. Then 2μL of the protein sample solution was applied to each anchor spot. After 2 min, the remaining liquid was removed by absorption using a paper tissue.

### Mass spectrometry and database searching

Matrix assisted laser desorption/ionization time-of-flight (MALDI-TOF) MS analysis was performed using an upgraded Reflex II MALDI-TOF MS (Bruker-Franzen Analytik GmbH, Bremen, Germany) equipped with a two-stage electrostatic reflectron, a delayed extraction (time-lag-focusing) ion source, a high resolution reflector detector and a 2 GHz digitizer. The spectra were acquired in the reflection mode at an accelerating voltage of 20 kV. The mass spectra, acquired and analyzed using Bruker software, were initially calibrated by external calibration using a mixture of known peptides and later recalibrated using two auto digestion products of porcine trypsin as internal calibrants. The protein database search tool "MASCOT Peptide Mass Fingerprint" on the Matrix Science web site [46] was used to compare the monoisotopic m/z values of the tryptic fragments to those of known proteins in the NCBI database. A mass deviation of 100 ppm was tolerated and Homo sapiens was specified.

### Statistical analysis

Coefficient of variation (CV) was calculated (standard deviation (SD)/ Mean × 100) of the normalized protein spot densities from four replicate 2-D gels.

In the proteomic study a 2-fold increase or decrease of normalized protein quantities was taken into account.

## Competing interests

The authors declare that they have no competing interests.

## Authors' contributions

SFH carried out all the 2D gel experiments, mass spectrometry analysis, participated in results evaluation and drafted the manuscript.

MP participated in the 2D gel experiments, results evaluation and participated to the manuscript writing.

KB contributed with material and critical reading of the manuscript.

MJ contributed with material and participated in the design of the study.

PD conceived the study, participated in its design and coordination, results analysis and supervised the manuscript writing.
